# Alternative Natural Rubber Cross-Linking Utilizing a Disulfide-Containing Bismaleimide

**DOI:** 10.3390/polym17243302

**Published:** 2025-12-13

**Authors:** Anureet Kaur, Maria Tucker, Keizo Akutagawa, Biqiong Chen, James J. C. Busfield

**Affiliations:** 1 School of Engineering and Materials Science, Queen Mary University of London, London E1 4NS, UKk.akutagawa@qmul.ac.uk (K.A.); 2 Department of Chemistry, University of Liverpool, Crown Street, Liverpool L69 7ZD, UK; biqiong.chen@liverpool.ac.uk

**Keywords:** bismaleimide, natural rubber, cross-linking, disulfide

## Abstract

This study explores a disulfide-selective cross-linking strategy for natural rubber (NR) to formulate elastomeric materials with engineering-relevant mechanical properties. A disulfide-containing bismaleimide (BIS) cross-linker was synthesized from cystamine and maleic anhydride and compounded with NR. Three formulations were prepared: control (no inhibitor), CuCl_2_-based, and copper(II) methacrylate (CuMA) based compounds, with BIS concentrations ranging from 3.55 to 8.88 phr. Rheological and mechanical testing revealed that CuCl_2_ formulations suffered from molecular degradation, poor thermal stability, and mechanical brittleness due to oxidative reactions in the absence of antioxidants. In contrast, CuMA-based compounds exhibited intermediate molecular weights prior to curing, stable thermal behavior, and improved mechanical properties, including enhanced torque and tensile strength, indicating effective cross-linking and partial recyclability. The control formulations also performed reasonably well but did not match the mechanical strength of conventional sulfur-vulcanized NR. The results demonstrate that metal coordination, particularly with CuMA, can modulate disulfide metathesis kinetics and offer a pathway to thermally triggered network rearrangement. Overall, CuMA emerges as a promising candidate for developing high-performance, recyclable rubber materials, while CuCl_2_ requires further stabilization strategies. This work establishes a baseline for future recyclability studies and advances the design of dynamic covalent networks in elastomers.

## 1. Introduction

Conventional sulfur vulcanization (CV) yields robust three-dimensional polysulfide cross-linked networks that provide the mechanical performance required by engineering elastomers but render the resulting materials essentially unrecyclable [1]. Efforts to recover value from vulcanized rubber have therefore focused on devulcanization and other recycling routes, yet selective and efficient reclaiming of cross-linked networks remains an as yet unfulfilled challenge for the rubber industry [2,3]. One promising route to reconcile durable performance with recyclability is the introduction of dynamic covalent linkages into the cross-link network. Disulfide bonds (S–S) are known to behave as dynamic covalent bonds, which have been reported to undergo a free radical disulfide metathesis reaction following a radical-mediated [2 + 1] mechanism, consisting of cleavage of the disulfide bond and its subsequent reforming [4], that can occur spontaneously [5] or be thermally initiated [6]. The latter category is particularly useful for the development of recyclable elastomers, as the exchange reaction necessary to rearrange the cross-linked network can be triggered only under specific conditions, retaining elastomeric behavior in a wide range of service conditions. The lability of S–S bonds relative to typical carbon–carbon single or carbon–carbon double bonds underpins their utility for this purpose [7]. Controlling the temperature, and therefore the timing, of disulfide metathesis is critical: facile exchange at ambient temperature compromises dimensional and mechanical stability, whereas an exchange that is triggerable only at processing temperatures enables reprocessing without loss of in-service performance. Recent work demonstrates that metal coordination, for example via copper(II) methacrylate (CuMA) or CuCl_2_, can be used to modulate disulfide metathesis kinetics and thus tune the rearrangement temperature window in sulfur-based vulcanizates, as the activation temperature is reached, the copper(II)-based complex catalysis can enable cross-over reactions between disulfide and polysulfides [8,9]. Notably, we have previously reported that optimizing a sulfur-based cure package with CuMA promotes a reversible disulfide/polysulfide network that can be recovered by thermal processing, thereby enabling recyclable sulfur-cured natural rubber formulations [1]. Motivated by these developments, the present study aimed to implement a different cross-linking architecture in an elastomer matrix by selectively introducing disulfide-only cross-links and evaluating the mechanical performance of the resulting pristine materials. The introduction of disulfide cross-links can only be achieved by either adjusting the sulfur/accelerator (S/A) ratio to >1 in a sulfur-based vulcanization—hence using the CV system, favoring the formation of polysulfide cross-links over monosulfides—or by introducing small organic molecules that contain disulfide functional groups. However, in CV-cured compounds that are characterized by a large number of polysulfides, once an S-S bond is cleaved and thiyl radicals are formed, they would statistically be keener to react with the NR chains, slowly shifting the cross-linked network to a semi-efficient system (SEV, where S/A ≈ 1 and 50% of the cross-links are polysulfides) or efficient system (EV, where S/A ≪ 1 and 80% of the cross-links are monosulfides) [10,11]. An example of disulfide-containing small organic molecules that can be used to cross-link natural rubber is disulfide-containing bismaleimides. These have been reported in the literature as capable of introducing self-healable and reprocessable properties in elastomers. Kitagawa et al. synthesized Jaffamine-derived polymers using bis(2-maleimidoethyl) disulfide and 4-aminophenyl disulfide as cross-linkers, achieving self-healing efficiencies higher than 90% after reprocessing at 180 °C [12]. Sim et al. cross-linked poly(furfuryl methacrylate) (PFMA) brushes with a disulfide-containing bismaleimide via the Diels–Alder (DA) reaction at 70 °C, where these could be decross-linked at 110 °C via the retro-Diels–Alder (rDA) reaction, and the S–S bond could be cleaved upon thermal or photo stimulus and regenerated through oxidative stimulus, offering another reversible decross-linking/cross-linking pathway for cross-linked PFMA brushes [13]. Usually, the rubber backbone is first grafted with maleic anhydride, which is then reacted with furfurylamine, and then, through DA, the bismaleimide molecule is introduced as a cross-linker [14]. More generally, bismaleimides to be introduced in polymers as cross-linkers require a furan functional group to react through the DA pathway [14,15,16]. However, grafting of maleic anhydride has been reported in the literature via application of shearing action in internal mixers and heat [17,18]. Following a similar approach, this work reports the cross-linking of NR with disulfide-containing bismaleimide via mixing in a micro compounder, followed by compression molding.

**Figure 1:**
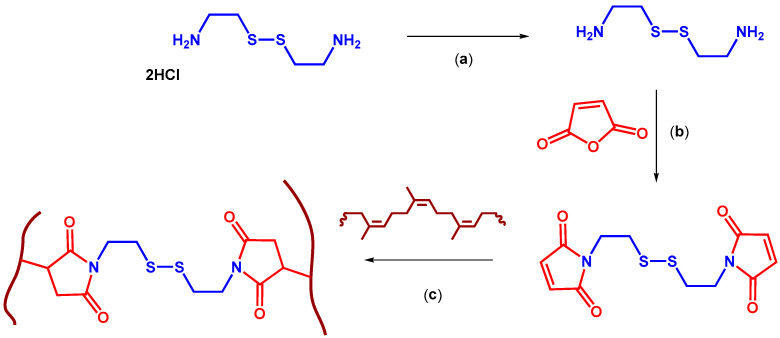
Schematic representation of rubber preparation. Conditions: (**a**) cystamine dihydrochloride, deionized water, NaOH, 12 h, room temperature; (**b**) 1. maleic anhydride, cystamine, toluene, 1 h, 30 °C, 2. hexamethyldisilazane, ZnBr_2_, 12 h, 85 °C; (**c**) 1,1′-(disulfanediylbis(ethane-2,1-diyl))bis(1H-pyrrole-2,5-dione), NR, mixing in the micro compounder, followed by curing with hot press for the time required to reach 90% of maximum torque, *t*_90_, at 180 °C.

This work reports the pre-synthesis of a disulfide-containing bismaleimide (BIS) cross-linker, starting from desalination of cystamine dihydrochloride with subsequent reaction of the resulting cystamine with maleic anhydride, followed by compounding with NR, as shown in Figure 1. Three formulations were prepared: (i) control compounds without inhibitors, (ii) CuCl_2_ compounds, and (iii) CuMA compounds. Copper(II) complexes act as catalysts; however, rather than lowering the activation energy, they increase the activation energy threshold to enable disulfide metathesis. Therefore, in this work, copper(II) compounds are referred to as inhibitors. In all three formulations, BIS concentration varied from 3.55 to 8.88 phr. Rheological cure behavior and standard mechanical tests were used to define cure parameters and to establish baseline tensile/elastic properties of the pristine compounds. This paper, therefore, (i) describes the design and processing of a disulfide-selective cross-linking strategy to form the intended dynamic and controlled cross-linked network, and (ii) reports the mechanical performance of the pristine materials as a baseline for any future recyclability studies. The results establish whether the selective introduction of disulfide cross-links and the incorporation of metal coordination agents can produce materials with engineering-relevant mechanical properties while offering a route to thermally trigger network rearrangement under controlled conditions. No additional accelerators or antioxidants were included in the formulations, as the primary objective of this study is to isolate and evaluate the specific influence of copper(II) compounds on disulfide-based cross-linking. Introducing accelerators or antioxidants could have introduced competing effects, such as altering cure rates or suppressing oxidative pathways, which would confound interpretation of the copper-mediated inhibition mechanism. By excluding these additives, we ensured that the observed changes in rheological and mechanical behavior could be attributed solely to the presence and coordination chemistry of the copper(II) species.

## 2. Materials and Methods

### 2.1. Materials

Natural rubber (NR, SMR CV60 grade) was purchased from the Tun Abdul Razak Research Centre (Hertford, UK). Maleic anhydride (Man, purity ≥ 98%, technical grade) was purchased from VWR International (Leicestershire, UK). Copper(II) chloride (CuCl_2_; purity ≥ 98%), hydrochloric acid (HCl; 37%, ACS reagent), hexamethyldisilazane (purity 99%), Chloroform-d (CDCl_3_; 99.8 atom % D), methanol (purity ≥ 99.8%, ACS reagent), and dichloromethane (purity ≥ 99.9%, ACS reagent) were purchased from Sigma-Aldrich Co Ltd. (Dorset, UK). Cystamine dihydrochloride (Cys; purity 97%), sodium hydroxide (purity 98%), copper(II) methacrylate (CuMA, technical grade), zinc bromide (purity 98%, anhydrous), sodium hydrogen carbonate (purity 99%), and anhydrous sodium sulfate (purity 99%) were purchased from Fisher Scientific (Loughborough, UK). Toluene (purity ≥ 99.9%, HPLC grade) and tetrahydrofuran (THF; purity ≥ 99.9%, inhibitor free, HPLC grade) were purchased from Honeywell International Inc. (Bracknell, UK).

In the present work, different rubber formulations with and without Cu(II) inhibitors were tested in different conditions, before and after curing and recycling. The terminology adopted to refer to the formulations and blends in specific conditions is as follows:

- NR/BIS-X: formulations without inhibitors, acting as control formulations, with X referring to phr of Bismaleimide introduced; generally called Control formulations;
- NR/BIS-X/CuMA: formulations with CuMA as inhibitor, with X referring to phr of Bismaleimide introduced; CuMA is equimolar to Bismaleimide;
- NR/BIS-X/CuCl_2_: formulations with CuCl_2_ as inhibitor, with X referring to phr of Bismaleimide introduced; CuCl_2_ is equimolar to Bismaleimide;
- Compound: something that has been mixed, but not cured;
- Cured compound: new cured compound;
- Recycled compound: recycled cured compound.

### 2.2. Preparation of Cystamine

Sodium hydroxide (10.65 g, 266.430 mmol) was dissolved in 25 mL of deionized water and was added dropwise to an aqueous solution of cystamine dihydrochloride (30 g, 133.215 mmol, in 25 mL of deionized water), in an ice bath. The resulting solution was stirred for 1 h at room temperature. The obtained solution was transferred into a separatory funnel, and dichloromethane was added (20 mL × 4). The organic phase was dried over anhydrous sodium sulfate and filtered before drying under vacuum in a rotatory evaporator to obtain cystamine as a pale-yellow oily liquid (yield 29.9%). The resulting product was characterized by ^1^H and ^13^C NMR spectroscopy with Bruker Advance III 400 MHz. ^1^H NMR (CDCl_3_, 400 MHz): chemical shift δ = 1.21, 2.61, 2.85 ppm. ^13^C DEPTQ135 NMR (CDCl_3_, 100 MHz): chemical shift δ = 40.56, 42.51 ppm.

### 2.3. Synthesis of Bismaleimide Cross-Linker

A suspension of maleic anhydride (0.6641 g, 6.77 mmol) and cystamine (0.6846 g, 3.39 mmol) was prepared in 80 mL of toluene and stirred at 30 °C for 1 h. Subsequently, zinc bromide (1.524 g, 6.77 mmol) and hexamethyldisilazane (1.093 g, 6.77 mmol), dissolved in 15 mL of toluene, were added to the reaction mixture. The resulting suspension was heated to 85 °C and refluxed for 2 h. Reaction progress was monitored by thin-layer chromatography (TLC) using silica gel F254 plates on aluminum sheets and a dichloromethane:methanol (9:1) eluent system (product retention factor = 0.0678), and the reaction was considered complete once the reactant stains disappeared. Upon completion, the mixture was cooled to room temperature and quenched with 50 mL of 1 M hydrochloric acid. The organic layer was separated, and the aqueous layer was extracted twice with 50 mL portions of ethyl acetate. The combined organic extracts were washed with a saturated aqueous solution of sodium hydrogen carbonate and brine, then dried over anhydrous sodium sulfate. The solution was filtered and concentrated under reduced pressure using a rotary evaporator. The crude product was purified by crystallization, and the concentrated solution was placed in a refrigerator overnight. The resulting crystals were collected by filtration, washed with cold methanol, and dried under reduced pressure for 2 h. The resulting Bismaleimide (BIS) product, 1,1′-(disulfanediylbis(ethane-2,1-diyl))bis(1H-pyrrole-2,5-dione) (yield 48%), was characterized by ^1^H and ^13^C NMR spectroscopy. ^1^H NMR (CDCl_3_, 400 MHz): δ = 2.95, 3.88, 6.74 ppm. ^13^C DEPTQ135 NMR (CDCl_3_, 100 MHz): δ = 35.92, 36.81, 134.05 ppm.

### 2.4. Compound Preparation and Curing

NR/BIS, NR/BIS/CuMA, and NR/BIS/CuCl_2_ formulations are reported in Table 1. Compounds were prepared in an Xplore MC15 HT Micro Compounder. Mixing was performed at 100 °C, 100 rpm speed, and a maximum torque of 35 Nm. NR, the previously synthesized BIS cross-linker, and the copper inhibitor were added together and mixed for 3 min. The compounds were then molded in a manual hot press at 180 °C, applying a constant pressure of about 12 MPa, for a curing time equal to *t*_90_ determined using Monsanto Moving Die Rheometer (MDR) 2000 with the lower die moving at 1.66 Hz, which was different for each formulation, as reported in Table 1.

### 2.5. Gel Permeation Chromatography

The number average molecular weight ($\bar{M}$_n_), the weight average molecular weight ($\bar{M}$_w_), and the dispersity (*Ð*) of NR and each compound prior to vulcanization were determined by performing Gel Permeation Chromatography (GPC) using Agilent Technologies 1260 Infinity GPC/SEC system equipped with a refractive index (RI) detector. Calibration was carried out using polystyrene standards ($\bar{M}$_w_ range from 162 g/mol to 6,570,000 g/mol) from Agilent Technologies, Inc. (Santa Clara, CA, USA). Samples were prepared at least 48 h prior to the analysis by dissolving approximately 10 mg of sample in 5 mL of THF. The solutions were then filtered through a 0.2 μm PTFE syringe filter and transferred into GPC vials. Each run was performed by injecting 100 μL into the columns, kept at a constant temperature of 25 °C.

### 2.6. Dynamic Differential Scanning Calorimetry (DSC)

Dynamic DSC was conducted to determine the glass transition temperature, *T*_g_, of all uncured compounds and cured compounds. Both analyses were carried out using a TA Instruments DSC25. All samples were placed in Tzero Aluminium Hermetic pans, and the normalized heat flow was measured. The thermal behavior of the uncured samples was investigated by conducting heat–cool–heat experiments. This is shown as follows: (1) first heating ramp from 20 °C to 200 °C at a rate of 5 °C/min; (2) second cool ramp from 200 °C to 20 °C at a rate of 5 °C/min; (3) third heat ramp from 20 °C to 200 °C at a rate of 5 °C/min. For the cured samples the heat–cool–heat experiments were carried as follows: (1) first heating ramp from −90 °C to 250 °C at a rate of 5 °C/min; (2) second cool ramp from 250 °C to −90 °C at a rate of 5 °C/min; (3) third heat ramp from −90 °C to 250 °C at a rate of 5 °C/min. The *T*_g_ of the cured samples was calculated by evaluating the minimum point of the first derivative of the normalized heat flow plot.

### 2.7. Mechanical Characterization

Tensile testing until failure was carried out on cured compounds. Dumbbell shape samples were cut out using an ISO-37-4 [19] die cutter 24 h after curing was completed. Testing was carried out using an Instron 5967 machine equipped with a 1 kN load cell and pneumatic side action grips, using a rate of 1 mm/s, which corresponds to an approximate strain rate of 0.1/s. The width and the thickness were measured prior to the start of the test, while the initial length, *L*_0_, was measured after gripping the sample, ensuring the width of the sample remained constant in the gauge length. Uniaxial stress and strain were calculated using the following Equations (1) and (2):

(1) $$\sigma =\frac{F}{A_{0}}$$

(2) $$\varepsilon =\frac{∆L}{L_{0}}$$

where σ is the engineering stress, *F* is the measured force, *A*_0_ is the initial unstrained cross-sectional area, ε is the strain, and ∆L is the measured elongation.

### 2.8. Density

Density measurements were carried out using a Micromeritics AccuPyc II 1345 gas-displacement pycnometer (Micromeritics Instrument Corporation, Norcross, GA, USA) using the following settings: number of purges 10, purge fill pressure 134.45 kPa, number of cycles 10, cycle fill pressure 134.45 kPa, equilibration rate 3.45 kPa min^−1^, and chamber size 1 cm^3^.

## 3. Results and Discussion

**Figure 2:**
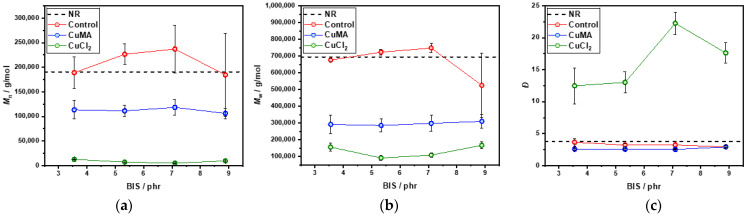
GPC analysis comparison between NR, Control, CuMA, and CuCl_2_ formulations: (**a**) ${\bar{M}}_{n}$ results, (**b**) ${\bar{M}}_{w}$ results, (**c**) *Đ* results.

During the mixing stage, rubber compounds may undergo molecular degradation due to mechanical and thermal stresses. Specifically, excessive mastication in the micro compounder can reduce $\bar{M}$_n_ and ${\bar{M}}_{w}$, while elevated temperatures may promote premature chemical interactions among formulation components. Disulfide metathesis reactions are particularly favored under these conditions, potentially leading to exchange reactions prior to curing. Figure 2a,b presents the $\bar{M}$_n_ and $\bar{M}$_w_ values respectively following a 3 min mixing period. The control formulation exhibits $\bar{M}$_n_ and $\bar{M}$_w_ values comparable to raw NR, with NR/BIS-8.88 showing lower $\bar{M}$_w_ (500,000 g/mol) than NR (700,000 g/mol), while $\bar{M}$_n_ remains in the range of 180,000–240,000 g/mol. In contrast, the CuCl_2_ formulation displays significantly reduced $\bar{M}$_n_ and $\bar{M}$_w_ (10,000–20,000 g/mol and 100,000–150,000 g/mol, respectively), indicating poor compatibility with NR and suggesting substantial chain scission during processing. CuMA samples show intermediate $\bar{M}$_n_ and $\bar{M}$_w_ values (100,000–110,000 g/mol and 300,000–310,000 g/mol), which remain suitable for further processing. Additionally reported in Figure 2c, *Đ* values for NR, Control, and CuMA formulations range between 3 and 5, indicating relatively uniform chain lengths.

**Figure 3:**
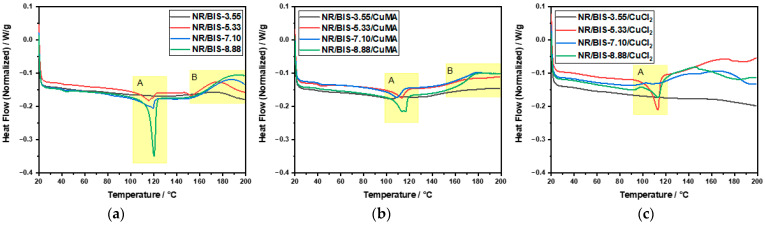
DSC analysis of uncured samples with peak A representing bismaleimide crystallization, and peak B indicating NR-cross-linker interaction: (**a**) Control, (**b**) CuMA, and (**c**) CuCl_2_ formulations.

**Figure 4:**
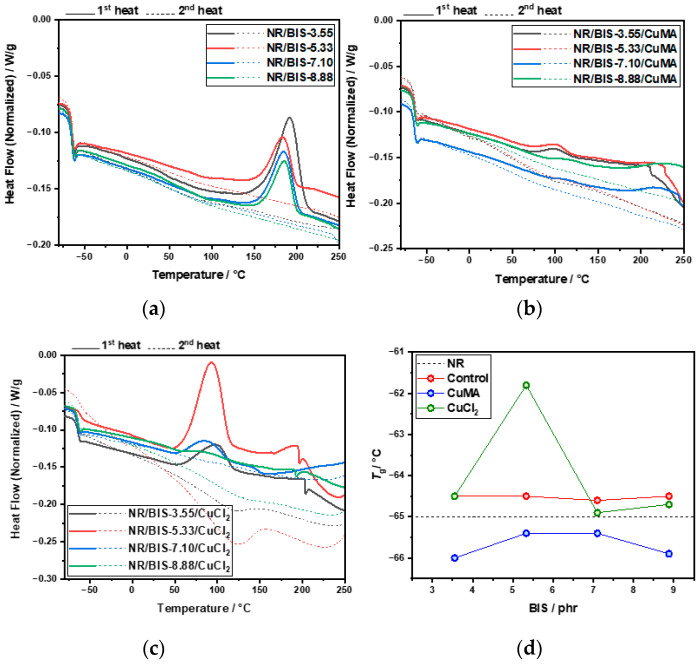
DSC analysis of cured samples: (**a**) Control, (**b**) CuMA, and (**c**) CuCl_2_ formulations. (**d**) *T*_g_ of all samples.

**Figure 5:**
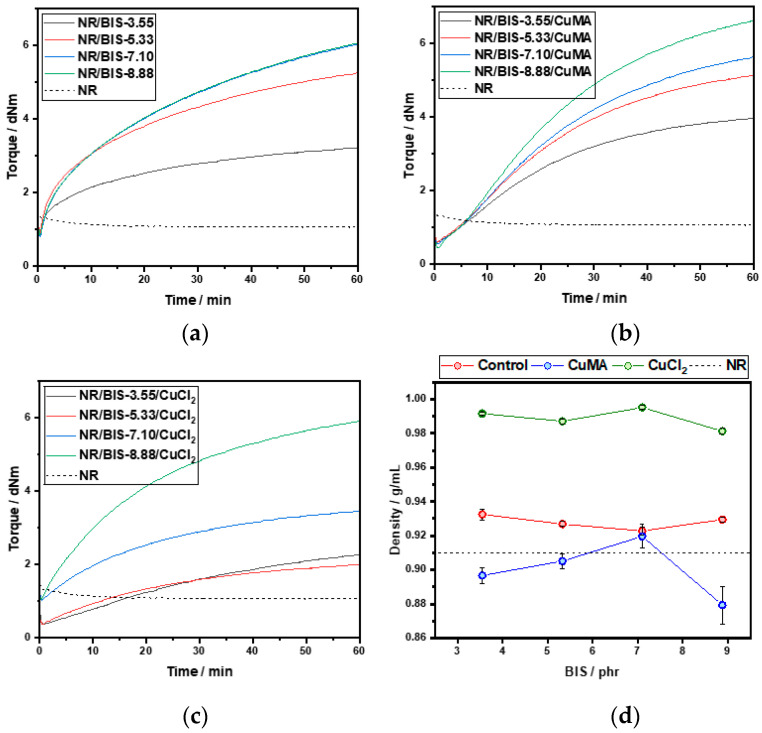
Rheology and density measurements. MDR analysis at 180 °C of (**a**) Control, (**b**) CuMA, and (**c**) CuCl_2_ formulations. (**d**) Density vs. BIS concentration on the *x*-axis.

**Figure 6:**
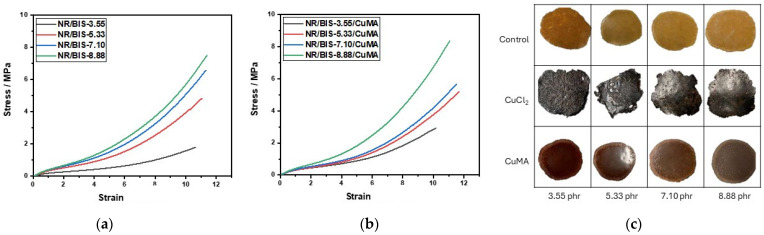
Tensile test analysis of (**a**) Control and (**b**) CuMA formulations. (**c**) Control, CuCl_2_, and CuMA samples after vulcanization.

**Figure 7:**
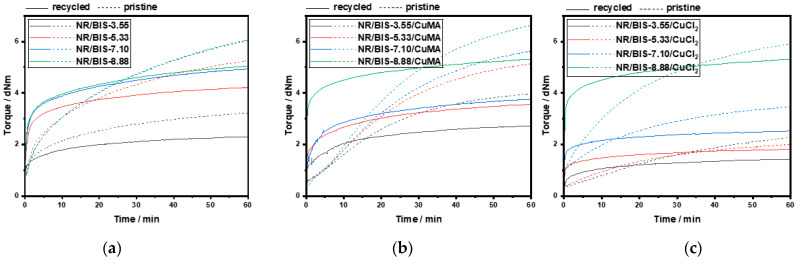
Rheology comparison between pristine and recycled samples: (**a**) Control, (**b**) CuMA, and (**c**) CuCl_2_.

However, CuCl_2_ formulations exhibit much broader *Đ* values between 11 and 23, further supporting the hypothesis of polymer degradation and incompatibility in the absence of antioxidants. Overall, the molecular weights of CuMA and CuCl_2_ compounds are lower than those of NR and Control compounds, primarily due to the mixing conditions of 100 °C at 100 rpm, and an additional 3 min mixing step required for incorporating the copper(II) inhibitors, which was not performed for the control formulation. DSC analysis was performed to determine the thermal behavior and curing characteristics of the uncured and cured rubber formulations. Dynamic DSC thermograms of uncured Control samples are reported in Figure 3a. Two distinct peaks are revealed: peak A between 115 °C and 120 °C, corresponding to the recrystallization of bismaleimide, and peak B (140–150 °C), associated with the reaction between NR and the cross-linker. As the cross-linker concentration increased from 3.55 to 8.88 phr, peak B shifted to higher temperatures, indicating delayed reaction onset. A curing temperature range of 170–190 °C was selected to accommodate all concentrations. DSC thermograms of CuMA samples reported in Figure 3b exhibited similar dual-peak behavior, with peak A representing bismaleimide crystallization, and peak B indicating NR-cross-linker interaction. The optimal curing temperature was again determined to be 170–190 °C. In contrast, CuCl_2_ samples reported in Figure 3c showed irregular thermograms with no distinct peaks, suggesting degradation and incompatibility with NR. The absence of antioxidants likely led to oxidative degradation, producing hydrochloric acid and chlorine gas through complex redox reactions. Cured samples were also analyzed via DSC. Control formulations reported in Figure 4a displayed a single peak in the first heating cycle, possibly due to residual cross-linker reactions or bismaleimide copolymerization, as in the second heating cycle, no peak appears in the same region. CuMA samples in Figure 4b showed minor peaks, indicating further cross-linker activity. CuCl_2_ samples in Figure 4c lacked distinct thermal transitions, reinforcing the conclusion of degradation. Glass transition temperatures, *T*_g_, for all formulations were consistent with unfilled NR (~−65 °C), as reported in Figure 4d. Rheological measurements were employed to assess and confirm the curing behavior and network formation of the rubber compounds at 180 °C. As reported in Figure 5, NR, lacking curatives, exhibited a baseline torque of 1.5 dNm due to physical entanglements rather than chemical cross-links. As shown in Figure 5a, increasing the concentration of cross-linkers in the Control samples led to higher torque values from 3 to 6 dNm. NR/BIS-7.10 demonstrated optimal performance, with torque values nearly matching those of NR/BIS-8.88, suggesting efficient cross-link formation. CuMA formulations reported in Figure 5b exhibited a consistent increase in torque with cross-linker concentration going from 4 to 6.5 dNm. NR/BIS-8.88/CuMA showed the highest torque values, indicating robust and stable cross-link networks. CuCl_2_ formulations reported in Figure 5c also showed increasing torque with higher cross-linker concentrations, going from 2 to 6 dNm. However, inconsistencies were observed, such as lower torque in NR/BIS-5.33/CuCl_2_ compared with NR/BIS-3.55/CuCl_2_, suggesting degradation. These results suggest that CuMA formulations are promising candidates for achieving high mechanical performance, while CuCl_2_ formulations suffer from degradation and poor network stability. All formulations exhibit short or negligible scorch times, which could potentially hinder mold flow. However, full mold filling was achieved because the compounds were processed under controlled conditions that maintained sufficient mobility before significant network formation occurred. The marching cure behavior of these systems ensures that cross-linking progresses gradually rather than instantaneously; initial viscosity remains low enough for the material to flow and completely fill the mold before torque rises substantially. This dynamic curing characteristic is typical of disulfide-based systems, where bond exchange occurs progressively rather than locking the network immediately. Density measurements, presented in Figure 5d, were conducted to assess the structural integrity and packing efficiency within the rubber matrix. Among the formulations, CuCl_2_ samples exhibited the highest density values between 0.98 and 1.00 g/mol, which suggests significant degradation. In contrast, CuMA formulations reported the lowest density values between 0.87 and 0.92 g/mol. This reduction may be attributed to the bulkier nature of the CuMA inhibitor, which could hinder tight packing and reduce the overall material density. Control samples reported higher density values compared with unfilled NR (0.91 ± 9.1 × 10^−4^ g/mL), suggesting minimal structural disruption and effective cross-linking. Tensile testing was conducted on the cured rubber compounds to evaluate their mechanical performance, as shown in Figure 6. Due to extensive degradation during hot pressing, the CuCl_2_ samples produced were too brittle to grip for tensile testing, highlighting their poor thermal and chemical stability in the absence of antioxidants. In contrast, the Control formulations reported in Figure 6a demonstrated a clear trend of increasing tensile strength with higher cross-linker concentrations. The maximum tensile stress recorded for Control NR/BIS-8.88 was 7.5 MPa, with a corresponding tensile strain of 11.5. Similarly, CuMA samples in Figure 6b exhibited comparable mechanical behavior, reaching a maximum tensile stress for NR/BIS-8.88/CuMA of 8.4 MPa and tensile strain of 11.1. The tensile strength is directly correlated with the density of cross-links formed during curing. Both Control and CuMA samples showed promising results, although they remain significantly below the tensile strength of conventionally sulfur-vulcanized unfilled NR, which typically reaches ~16 MPa [1]. This reduction in strength suggests that while bismaleimide-based cross-linking is effective, further optimization is required to match commercial compounds. To explore the recyclability of the materials, recycled samples were evaluated using rheology as a preliminary indicator of network retention, as torque values can provide insight into the extent of cross-link rearrangement and degradation upon reprocessing. As reported in Figure 7a, the recycled torque values of the Control samples ranged from 2 to 6 dNm. All samples showed reduced torque values compared with the respective pristine samples, suggesting partial retention of the cross-linked network and potential for limited recyclability. CuMA samples reported in Figure 7b showed better performance upon reprocessing, with torque values ranging from 2.5 to 4.5 dNm. NR/BIS-8.88/CuMA formulation retained the highest torque of 4.6 dNm, suggesting that the disulfide-based cross-links formed in this system are more resistant to breakdown and may allow for limited reprocessability. CuCl_2_ samples reported in Figure 7c exhibited similar behavior, with reprocessed torque values ranging from 1 to 5.5 dNm.

## 4. Conclusions

The study demonstrates that rubber formulations are highly sensitive to processing conditions and component compatibility. CuCl_2_-based compounds exhibit significant main chain scission and degradation, poor thermal stability, and mechanical brittleness, largely due to oxidative reactions in the absence of antioxidants. In contrast, CuMA outperformed CuCl_2_, likely because the methacrylate ligand provides a more stable coordination environment for copper(II) ions, thereby reducing their propensity to induce uncontrolled oxidation. As a result, CuMA formulations maintain intermediate molecular weights, stable thermal behavior, and promising mechanical properties, including enhanced torque and tensile strength, suggesting effective cross-linking and partial recyclability. Control formulations also perform well, though both CuMA and Control systems fall short of the mechanical strength observed in conventional sulfur-vulcanized NR. While recyclability testing was planned as a subsequent phase of the project, experimental progress ceased before those assessments could be completed. As such, recyclability data are not reported here. However, the observed molecular characteristics and mechanical resilience of CuMA formulations strongly support further investigation into their recyclability. Future studies could explore thermal reprocessing, chemical depolymerization, or mechanical recovery strategies to validate and optimize the recyclability of these materials. Overall, CuMA emerges as a viable alternative for high-performance, recyclable rubber materials, while CuCl_2_ is unsuitable without further stabilization strategies.

## Figures and Tables

**Table 1:** NR/BIS, NR/BIS/CuMA, and NR/BIS/CuCl_2_ formulations and curing time.

Formulation	NR/phr	BIS/phr	CuMA/phr	CuCl_2_/phr	*t*_90_/min
NR/BIS-3.55	100	3.55			42
NR/BIS-3.55/CuMA	100	3.55	2.38		42
NR/BIS-3.55/CuCl_2_	100	3.55		1.37	49
NR/BIS-5.33	100	5.33			43
NR/BIS-5.33/CuMA	100	5.33	3.58		44
NR/BIS-5.33/CuCl_2_	100	5.33		2.06	45
NR/BIS-7.10	100	7.10			46
NR/BIS-7.10/CuMA	100	7.10	4.77		45
NR/BIS-7.10/CuCl_2_	100	7.10		2.74	43
NR/BIS-8.88	100	8.88			46
NR/BIS-8.88/CuMA	100	8.88	5.96		45
NR/BIS-8.88/CuCl_2_	100	8.88		3.43	44

## Data Availability

The original data presented in the study are openly available in the Queen Mary Research Online repository at https://qmro.qmul.ac.uk/xmlui/handle/123456789/114491 (accessed on 5 December 2025).

## References

[1] Kaur A., Fefar M.M., Griggs T., Akutagawa K., Chen B., Busfield J.J.C. (2024). Recyclable sulfur cured natural rubber with controlled disulfide metathesis. Commun. Mater..

[2] Markl E., Lackner M. (2020). Devulcanization Technologies for Recycling of Tire-Derived Rubber: A Review. Materials.

[3] Coran A.Y. (2003). Chemistry of the vulcanization and protection of elastomers: A review of the achievements. J. Appl. Polym. Sci..

[4] Nevejans S., Ballard N., Miranda J.I., Reck B., Asua J.M. (2016). The underlying mechanisms for self-healing of poly(disulfide)s. Phys. Chem. Chem. Phys..

[5] Rekondo A., Martin R., Ruiz de Luzuriaga A., Cabañero G., Grande H.J., Odriozola I. (2014). Catalyst-free room-temperature self-healing elastomers based on aromatic disulfide metathesis. Mater. Horiz..

[6] Kaur A., Gautrot J.E., Cavalli G., Watson D., Bickley A., Akutagawa K., Busfield J.J.C. (2021). Novel Crosslinking System for Poly-Chloroprene Rubber to Enable Recyclability and Introduce Self-Healing. Polymers.

[7] Imbernon L., Oikonomou E.K., Norvez S., Leibler L. (2015). Chemically crosslinked yet reprocessable epoxidized natural rubber via thermo-activated disulfide rearrangements. Polym. Chem..

[8] Xiang H.P., Qian H.J., Lu Z.Y., Rong M.Z., Zhang M.Q. (2015). Crack healing and reclaiming of vulcanized rubber by triggering the rearrangement of inherent sulfur crosslinked networks. Green Chem..

[9] Xiang H.P., Rong M.Z., Zhang M.Q. (2016). Self-healing, Reshaping, and Recycling of Vulcanized Chloroprene Rubber: A Case Study of Multitask Cyclic Utilization of Cross-linked Polymer. ACS Sustain. Chem. Eng..

[10] Kong Y., Chen X., Li Z., Li G., Huang Y. (2023). Evolution of crosslinking structure in vulcanized natural rubber during thermal aging in the presence of a constant compressive stress. Polym. Degrad. Stab..

[11] Wang M., Wang R., Chen X., Kong Y., Huang Y., Lv Y., Li G. (2022). Effect of non-rubber components on the crosslinking structure and thermo-oxidative degradation of natural rubber. Polym. Degrad. Stab..

[12] Kitagawa S., Ozawa M., Shibata M. (2023). Self-healing and reprocessable bismaleimide-diamine thermosets containing disulfide linkages. Polymer.

[13] Sim X.M., Wang C.-G., Liu X., Goto A. (2020). Multistimuli Responsive Reversible Cross-Linking–Decross-Linking of Concentrated Polymer Brushes. ACS Appl. Mater. Interfaces.

[14] Polgar L.M., Cerpentier R.R.J., Vermeij G.H., Picchioni F., Duin M.v. (2016). Influence of the chemical structure of cross-linking agents on properties of thermally reversible networks. Pure Appl. Chem..

[15] Gevrek T.N., Sanyal A. (2021). Furan-containing polymeric Materials: Harnessing the Diels-Alder chemistry for biomedical applications. Eur. Polym. J..

[16] Post C., van den Tempel P., Herrera Sánchez P., Maniar D., Bose R.K., Voet V.S.D., Folkersma R., Picchioni F., Loos K. (2025). Thermoreversible Diels–Alder Cross-Linking of BHMF-Based Polyesters: Synthesis, Characterization and Rheology. ACS Sustain. Chem. Eng..

[17] Hayeemasae N., Sensem Z., Sahakaro K., Ismail H. (2020). Maleated Natural Rubber/Halloysite Nanotubes Composites. Processes.

[18] Ujianto O., Noviyanti R., Wijaya R., Ramadhoni B. (2017). Effect of maleated natural rubber on tensile strength and compatibility of natural rubber/coconut coir composite. IOP Conf. Ser. Mater. Sci. Eng..

[19] (2024). Rubber, Vulcanized or Thermoplastic—Determination of Tensile Stress-strain Properties.

